# Biochemical recurrence prediction after robot-assited radical prostatectomy (BCR-PRARP)

**DOI:** 10.1016/j.heliyon.2024.e41031

**Published:** 2024-12-07

**Authors:** Tanan Bejrananda, Kiyoshi Takahara, Dutsadee Sowanthip, Tomonari Motonaga, Kota Yagi, Wataru Nakamura, Masanobu Saruta, Takuhisa Nukaya, Masashi Takenaka, Kenji Zennami, Manabu Ichino, Hitomi Sasaki, Makoto Sumitomo, Ryoichi Shiroki

**Affiliations:** a Division of Urology, Department of Surgery, Faculty of Medicine, Prince of Songkla University, Hat Yai, Songkhla, Thailand; b Department of Urology, Fujita Health University School of Medicine, Toyoake, Aichi, Japan; c Division of Urology, Department of Surgery, Faculty of Medicine, Chulalongkorn University, King Chulalongkorn Memorial Hospital, The Thai Red Cross Society, Bangkok, Thailand

**Keywords:** Nomograms, Prostatectomy, Prediction, Biochemical recurrence, Robot-assisted

## Abstract

**Objective:**

This study aimed to establish a robust predictive model for biochemical recurrence (BCR) in patients with prostate cancer who underwent robot-Assisted Radical Prostatectomy.

**Material and methods:**

A cohort of 1700 patients who underwent robot-assisted radical prostatectomy (RARP) for prostate cancer between August 2009 and December 2022 was included. BCR was defined as two consecutive PSA levels exceeding 0.2 ng/mL post-radical prostatectomy. Cox proportional hazards regression identified predictive variables for BCR. Subsequently, pathologic T stage, PSA level, positive surgical margin, extraprostatic extension, and seminal vesicle involvement were retained. A nomogram was constructed using R software to predict BCR. The model was evaluated using the C-index and calibration curves.

**Results:**

A total of 161 instances of BCR were observed during a median follow-up of 61.0 months (range, 12–162 months). The 5-year BCR-free survival rate for the cohort was 25 %. Univariate analysis demonstrated significant associations between BCR and PSA, clinical T stage, biopsy Gleason score, D'Amico risk classification, pathologic T stage, pathologic Gleason score, extraprostatic extension, seminal vesicle invasion, and positive surgical margins. Multivariate analysis identified high PSA ≥20 ng/mL (HR: 1.93; p = 0.034), pathologic T stage 3–4 (HR: 1.89; p < 0.001), pathologic Gleason score 8–10 (HR: 5.43; p < 0.001), extraprostatic extension (HR: 1.41; p < 0.001), seminal vesicle involvement (HR: 1.92; p = 0.018), and positive surgical margin (HR: 2.73; p < 0.001) as independent predictors of BCR. The new model exhibited a C-index of 0.743 (95 % confidence interval: 0.741–0.745).

**Conclusion:**

The developed nomogram accurately predicts the likelihood of BCR-free status within 3 years following RARP. This allows for tailored follow-up strategies, optimizing resource allocation, and holds significant clinical utility, warranting broader implementation and further research.

## 1 Introduction

Radical prostatectomy (RP) is the primary treatment for prostate cancer. However, a considerable percentage of patients (approximately 15–45 %) experience biochemical recurrence (BCR) after the procedure [1]. Accurately predicting the timing of postoperative BCR is crucial in determining subsequent treatment approaches. In 1999, a predictive model was established incorporating various factors such as preoperative prostate-specific antigen (PSA) levels, pathological Gleason scores (GS), extracapsular extension (ECE), seminal vesicle invasion (SVI), lymph node invasion, and positive surgical margins (PSM). This model remains a valuable tool for determining the appropriate treatment strategies. Kattan developed a nomogram capable of predicting the BCR in patients who underwent RP for prostate cancer. This nomogram was subsequently validated at multiple centers in the United States [[2], [3], [4], [5]]. Several prediction models inspired by the Kattan postoperative nomogram were subsequently introduced [[6], [7], [8]].

The primary focus of these models is to predict the probability of long-term BCR. However, it is noteworthy that approximately two-thirds of BCR cases manifest within the first 3 years after undergoing RP [9,10]. Consequently, a model that can accurately forecast the BCR probability within this critical timeframe holds greater significance than previous models.

Contrastingly, previous models have predominantly relied on clinical or pathological data obtained during the perioperative period, neglecting the inclusion of short-term postoperative PSA levels acquired during follow-up. Nonetheless, PSA is a serum marker requiring regular monitoring after RP. Previous studies have demonstrated the potential importance of short-term postoperative PSA levels in predicting BCR. [[11], [12], [13]] In theory, prostatectomy completely eliminates prostate tumors, leading to a decline in PSA levels to an undetectable range within 6 weeks. However, persistent elevated PSA levels after prostatectomy suggest the presence of residual microtumor foci that require further management. Hence, this study aimed to develop a novel nomogram prediction model based on postoperative elevated PSA level recurrence.

This model sought to enhance the accuracy of predicting the probability of BCR after robot-assited prostatectomy, simplifying existing clinical follow-up strategies and aiding in the early prediction of patients at high risk of BCR.

## 2 Materials and methods

### 2.1 Study participants

A total of 1700 patients from University-based hospital who underwent robot-assisted radical prostatectomies between August 2009 and December 2022 were included in this study. Data were collected prospectively and entered into an electronic database by trained personnel. Regular quality control checks were performed to ensure the accuracy of the data. With a median follow-up of 61 months (range, 12–162 months). Consider excluding cases with observation periods less than one year from the analysis.

### 2.2 Variable definition

The primary focus of this study was to investigate BCR as a primary outcome. In this study, BCR was defined as the occurrence of two consecutive elevations in PSA levels after undergoing RP, with both measurements surpassing 0.2 ng/mL. However, it is important to note that there is currently no universally accepted international standard for the PSA threshold that indicates BCR following RP. Since the publication of the consensus by European urological experts in 2004, two distinct criteria have been widely used, and more researchers have recognized consecutive PSA levels above 0.2 ng/mL [14].

The threshold of 0.2 ng/mL was selected based on the consensus from the European Urological Association (EAU) guidelines. While we recognize the absence of a universally accepted threshold, the value of 0.2 ng/mL is commonly adopted by researchers and clinicians in numerous studies [15].

Due to the improved lymph node yield, better staging, and theoretical improvement in the control of micro-metastatic disease, guidelines have supported the use of pelvic lymph node dissection (PLND) in patients deemed to be at intermediate or high risk of lymph node involvement and cases with lymph node involvement were excluded. Neoadjuvant conventional androgen deprivation therapy agents (luteinizing hormone–releasing hormone (LHRH) analogues with or without first generation antiandrogens) in the setting of neoadjuvant therapy before radical prostatectomy for patients with clinically localized high risk-prostate cancer [goserelin monotherapy, cyproterone monotherapy and combination therapy with either leuprolide plus flutamide or goserelin plus flutamide].

### 2.3 Statistical analysis

We used Cox regression analysis to identify the risk factors associated with BCR. The univariate regression analysis identified important variables, which were subsequently incorporated into the multivariate analysis. The multivariate analysis employed a backward stepwise regression approach, leading to the exclusion of non-significant variables. The remaining significant variables were utilized in constructing the nomogram model, employing the regression modeling strategy package in R software (Windows version 4.3.1; http://www.r-project.org/). The consistency index (C-index) of the nomogram model was computed, and a calibration curve was generated to compare predicted values from the nomogram with actual values. Internal validation was performed via 500 iterations of repeated sampling using the bootstrap method, while external validation utilized data from the validation group. The validation cohort was selected from a separate institution using the same inclusion criteria, ensuring that the group represents a comparable patient population.

## 3 Results

The clinical and pathological characteristics of patients are shown in Table 1.

**Table 1:** Clinical and pathological Features of patients.

	Total
**Total**	1700
**Age, years**	
Median (IQR)	67 (63,71)
**Age, number**	
<65	561 (33.0)
≥65	1137 (66.9)
Unknown	2 (0.1)
**PSA(ng/mL)**	
Median (IQR)	7.5 (5.5–10.7)
**PSA (ng/mL)**	
<10	1194 (70.2)
10–19.00	391 (23.0)
≥20	112 (6.6)
Unknown	3 (0.2)
**BMI (Kg/m**^**2**^**)**	
<18.5	38 (2.2)
18.5–22.9	635 (37.4)
23–24.9	491 (28.9)
25–29.9	492 (28.9)
≥30	34 (2.0)
Unknown	10 (0.6)
**Clinical T stage**	
1–2b	1287 (75.7)
2c	289 (17.0)
3–4	120 (7.1)
Unknown	4 (0.2)
**Clinical Gleason score**	
6	393 (23.1)
7	872 (51.3)
8–10	431 (25.4)
Unknown	4 (0.2)
**Preoperative potency**	
No erection	660 (38.8)
Erection	548 (32.2)
Penetrate	462 (27.2)
Unknown	30 (1.8)
**D'Amico risk classification**	
High	698 (41.1)
Intermediate	769 (45.2)
Low	229 (13.5)
Unknown	4 (0.2)
**Neoadjuvant treatment**	
None	1090 (64.1)
ADT	166 (9.7)
Anti-A	178 (10.5)
NACHT	246 (14.5)
Unknown	20 (1.2)
**Nerve-sparing technique**	
None	497 (29.2)
Bilateral	207 (12.2)
Unilateral	996 (58.6)
**Pathologic T stage**	
1–2b	393 (23.1)
2c	1026 (60.4)
3–4	281 (16.5)
**Pathologic Gleason score**	
6	167 (9.8)
7	1191 (70.1)
8–10	342 (20.1)
**Surgical margin**	
Negative	1354 (79.6)
Positive	346 (20.4)
**Extraprostatic extension**	
Negative	1,469 (86.4)
Positive	231 (13.6)
**Seminal vesicle involvement**	
Negative	1606 (94.5)
Positive	94 (5.5)

Independent risk factors associated with BCR were identified By applying Cox regression to the clinical and pathological information of the modeling group (Table 2). Univariate analysis revealed that PSA, clinical T stage, biopsy Gleason score, D'Amico risk classification, pathological T stage, pathologic Gleason score, extraprostatic extension, SVI, and positive surgical margins were independent risk factors for BCR. Multivariate analysis showed a significant association with BCR. Multivariate analysis showed that high PSA ≥20 ng/mL (HR: 1.93; p = 0.034), pathological T stage 3–4 (HR: 1.89; p < 0.001), pathological Gleason score 8–10 (HR: 5.43; p < 0.001), extraprostatic extension (HR: 1.41; p < 0.001), SVI (HR: 1.92; p = 0.018), and positive surgical margin (HR: 2.73; p < 0.001) were independent predictors of BCR considered the independent risk factors and established in the final model.

**Table 2:** Independent predictors of BCR with Cox proportional hazards model.

Variables	Univariate analysis		Multivariate analysis	
	Hazard ratio (95 % CI)	P value	Hazard ratio (95 % CI)	P-value
**Age:**	0.83 (0.59–1.17)	0.289		
≥65 vs < 65				
**PSA (ng/mL)**				
<10	**Ref.**			
10–19.99	1.51 (1.03,2.19)	0.033	1.18 (0.79–1.76)	0.424
≥20	2.75 (1.65,4.59)	<0.001	1.93 (1.05–3.53)	0.034
**Clinical Gleason score**				
6	**Ref.**		**Ref.**	
7	2.77 (1.58,4.86)	<0.001	1.84 (0.87–3.9)	0.11
8–10	4.2 (2.35,7.53)	<0.001	1.81 (0.74–4.45)	0.195
**Clinical T stage**				
1–2b	Ref.			
2c	1.86 (1.25–2.76)	0.003	1.24 (0.66–2.3)	0.504
3–4	2.67 (1.61–4.43)	<0.001	1.71 (1.02–2.89)	0.043
**D'Amico risk classification**				
Low	Ref.		**Ref.**	
Intermediate	2.79 (1.26–6.2)	0.66	1.75 (0.75–4.07)	0.084
High	4.82 (2.2–10.56)	<0.001	2.16 (0.9–5.2)	0.196
**Pathologic T stage**				
1–2b	**Ref.**		**Ref.**	
2c	0.74 (0.48–1.13)	0.216	0.66 (0.42–1.03)	0.173
3–4	2.87 (1.82–4.51)	<0.001	1.89 (0.82–4.38)	<0.001
**Pathologic Gleason score**				
6	**Ref.**		**Ref.**	
7	3.61 (1.31–9.95)	0.011	2.74 (0.98–7.65)	0.054
8–10	8.67 (3.09–24.29)	<0.001	5.43 (1.89–15.57)	<0.001
**Extraprostatic extension**	2.72 (1.86–3.97)	<0.001	1.41 (1.02–2.18)	<0.001
Yes vs. No				
**Seminal vesicle involvement**	3.92 (2.4–6.39)	<0.001	1.92 (1.12–3.29)	0.018
Yes vs. No				
**Surgical margin**	3.42 (2.44–4.79)	<0.001	2.73 (1.87–3.98)	<0.001
Yes vs. No				

**Fig. 1:**
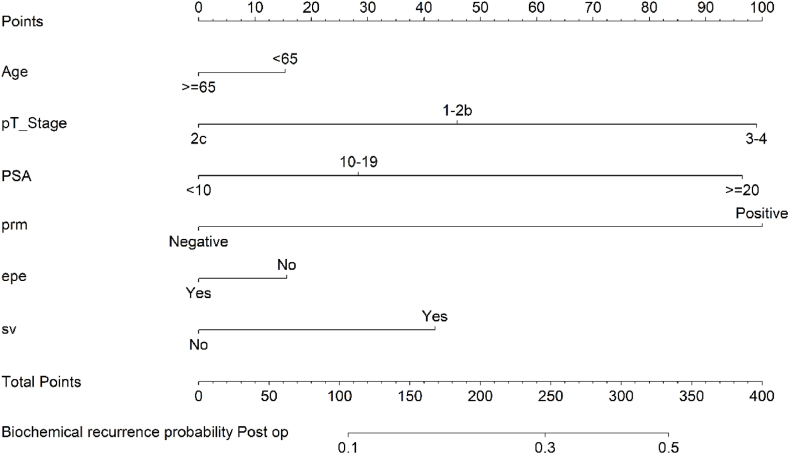
Nomogram predicting BCR after robot-assisted radical prostatectomy. A vertical line perpendicular to the horizontal axis was made according to the corresponding state of each predictor, and the intersection point between the vertical and upper score lines was the score of the predictor. The score of the five predictors was added to obtain the final score of the patient.

The nomogram model serves as a graphical representation of the underlying regression equation. To establish a scoring system, we assigned scores based on the magnitude of the regression coefficients for each independent variable. These scores were then allocated to different levels of each independent variable. Consequently, for every patient, a comprehensive score was computed, enabling the determination of the probability associated with the outcome time for that specific patient. This calculation was facilitated by employing a conversion function that links the score to the corresponding probability of the outcome (Fig. 1).

**Fig. 2:**
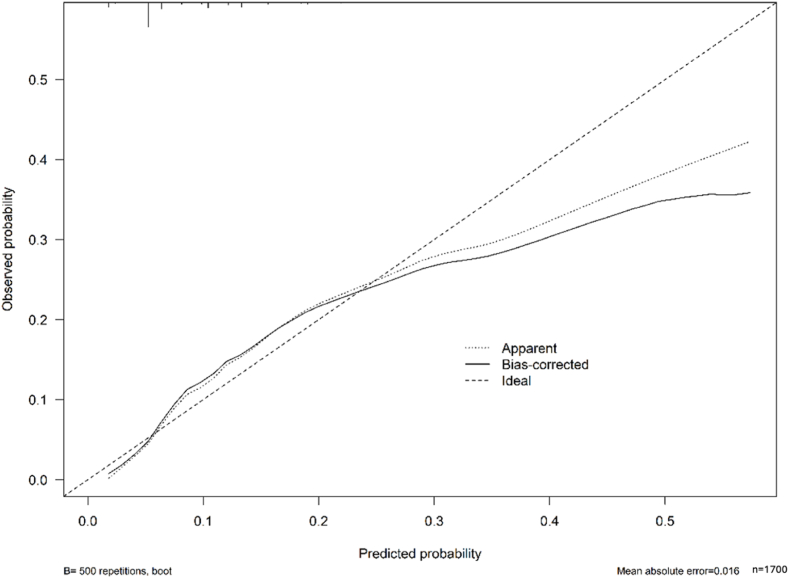
Calibration curve of the new model.

**Fig. 3:**
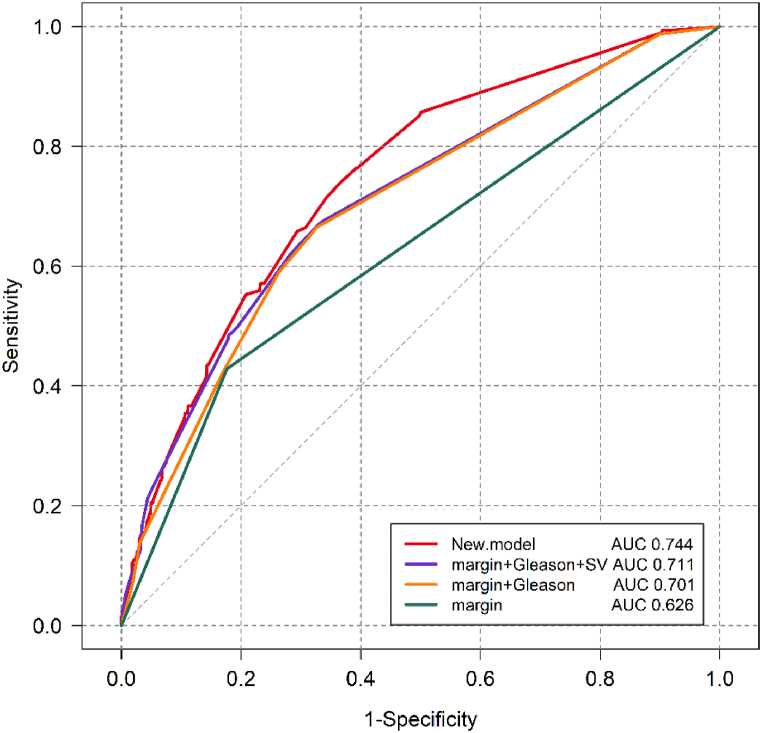
The ROC curve of the prediction model.

Internal validation of the prediction model showed that the C-index's accuracy was 0.743 (95 % confidence interval [CI]: 0.741–0.745). Calibration of the new model in the validation group also demonstrated good consistency between the predicted and actual values**,** indicating that the prediction model performed reasonably well in distinguishing individuals with varying outcome probabilities(Fig. 2). Moreover, the area under the receiver operating characteristic (ROC) curve (AUC) was 0.744. This indicates that the model has a significant accuracy in predicting BCR in this context (Fig. 3).

**Fig. 4:**
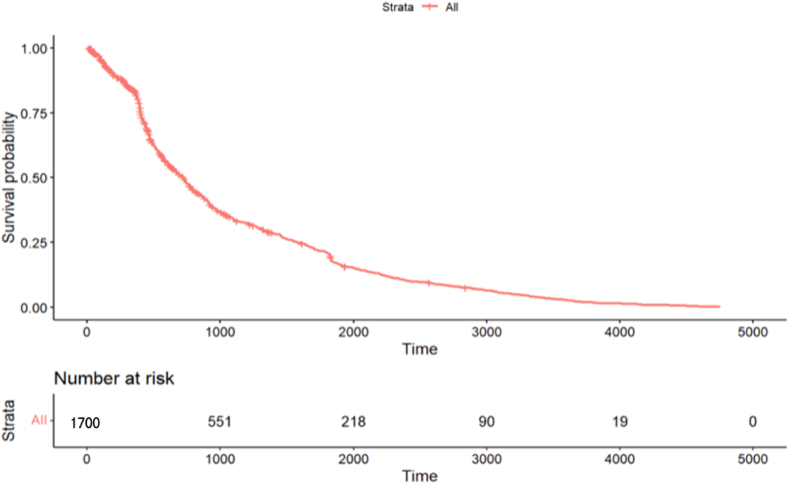
Kaplan-Meier survival curve of biochemical recurrence-free survival.

The BCR-free survival rate for the entire cohort at the median follow-up time was 25 %, and the 1-, 3-, and 5-year estimates were 90 %, 37.5 %, and 22.5 %, respectively. Fig. 4 shows the BCR-free survival curve after RARP.

## 4 Discussion

BCR is a crucial factor in determining whether a change in treatment strategy is necessary in patients who have undergone RP. Approximately 34 % of patients may progress to distant metastasis without additional radiotherapy or endocrine therapy after experiencing BCR [16]. Currently, a sole clinical approach is available for identifying the incidence of BCR. Nevertheless, research findings indicate that the rate of BCR within a decade after undergoing RP is 34.3 % [1]. Throughout the years, researchers have dedicated their efforts to developing a statistical model that can precisely forecast the likelihood of BCR by utilizing clinical and pathological data from patients. The postoperative nomogram introduced by Kattan et al., in 1999 is widely recognized as the most prominent model in this field [2]. Utilizing preoperative PSA levels and postoperative factors such as the Gleason score, ECE, SVI, and PSM, this nomogram demonstrated a high degree of effectiveness in estimating patients' probability of BCR. Subsequently, several scholars proposed a range of prediction models based on the foundation of the Kattan postoperative nomogram. These models incorporate specific variables that emphasize the robotic approach to enhance the overall prediction accuracy of the model [[6], [7], [8],^17^]. With efforts to enhance the model's prediction accuracy, the C-index was 0.743 (95 % CI: 0.741–0.745) indicating that the predictive model for BCR after robotic-assited prostatectomy performed well in discriminating between patients at different risk levels. These findings suggest that the model exhibits reasonably accurate ability to predict the occurrence of BCR within this population. Prediction models that amalgamate multiple indicators to anticipate disease occurrence or progression have been extensively used in various medical research domains. The “Transparent Reporting of a Multivariable Prediction Model for Individual Prognosis or Diagnosis” statement published in 2015 aimed to standardize the process of establishing prediction models [18]. Furthermore, Alba et al. specified standard statistical methods for constructing a prediction model in 2017 [19]. A reliable prediction model typically comprises two essential components: discrimination and calibration. Discrimination pertains to using an indicator to differentiate the risk of a specific event within a group, often assessed using the AUC or C-index. Calibration, another crucial evaluation index, reflects the agreement between predicted and actual values. Integrated Discrimination Improvement, proposed by Pencina et al., in 2008, is a statistical method used to compare two models [20]. However, as reported by Wessler et al., only 63 % of prediction model studies reported discrimination, and 36 % reported calibration [21]. The ROC curve and corresponding AUC offer valuable insights into the overall performance of the BCR prediction model, enabling clinicians and researchers to evaluate its predictive capabilities. These results indicate that the new model enhances the prediction performance, particularly in RARP.

Moreover, about the sentinel node technique and its relevance to pelvic lymph node dissection (PLND) in prostate cancer [[22], [23], [24]]. Unfortunately, data on variant histologies and aberrant growth patterns were not available for our cohort.According to the European Association of Urology guidelines, PSA levels are typically assessed every 6 months for the initial 3 years post-prostatectomy and yearly thereafter [25]. A limitation of this study is that the long time span of the study may introduce variation due to changing surgical techniques and case volumes. External validation using independent data is crucial and we have alsosentien performed external validation. The predictive nomogram model provides a user-friendly and precise means to estimate the likelihood of achieving a BCR-free status within a three-year timeframe for each individual patient. This empowers healthcare providers to tailor follow-up strategies specifically to each patient, effectively minimizing the unnecessary allocation of medical resources. This model holds significant clinical utility, offering a valuable tool for optimizing patient care. Its potential benefits warrant further dissemination and widespread adoption in clinical practice. However, as the data were obtained from a single center, data from other centers is needed for further validation.

## CRediT authorship contribution statement

**Tanan Bejrananda:** Writing – review & editing, Writing – original draft, Visualization, Validation, Supervision, Software, Resources, Project administration, Methodology, Investigation, Funding acquisition, Formal analysis, Data curation, Conceptualization. **Kiyoshi Takahara:** Writing – review & editing, Writing – original draft, Validation, Supervision, Project administration, Methodology, Investigation, Funding acquisition, Formal analysis, Data curation, Conceptualization. **Dutsadee Sowanthip:** Conceptualization. **Tomonari Motonaga:** Conceptualization. **Kota Yagi:** Conceptualization. **Wataru Nakamura:** Conceptualization. **Masanobu Saruta:** Conceptualization. **Takuhisa Nukaya:** Conceptualization. **Masashi Takenaka:** Conceptualization. **Kenji Zennami:** Conceptualization. **Manabu Ichino:** Conceptualization. **Hitomi Sasaki:** Conceptualization. **Makoto Sumitomo:** Conceptualization. **Ryoichi Shiroki:** Writing – review & editing, Writing – original draft, Supervision, Conceptualization.

## Informed consent

N/A.

## Approval of the research protocol by an Institutional Reviewer board

Approval number of research protocol by the Ethics Committee of the Fujita Health University Hospital (Approval No. HM19-265).

## Registry and the registration number of the study/trial

N/A.

## Animal studies

N/A.

## Declaration of competing interest

We, the undersigned authors of the manuscript titled "Biochemical Recurrence Prediction After Robot-Assisted Radical Prostatectomy (BCR-PRARP)," declare that:

1. We have no conflicts of interest to disclose, either financial or non-financial, in relation to the research, authorship, or publication of this article.

2. We have not received any financial support, funding, or sponsorship from any company, institution, or organization related to this study.

3. The research was conducted independently, and all data used in this study were collected and analyzed without influence or interference from external parties.

4. All co-authors have contributed significantly to the research, writing, and revision of this manuscript, and we have approved the final version of the manuscript for submission.

5. There are no personal relationships, affiliations, or associations with any organizations or individuals that could potentially bias the results or interpretation of this study.
